# An open, prospective cohort study of VV116 in Chinese participants infected with SARS-CoV-2 omicron variants

**DOI:** 10.1080/22221751.2022.2078230

**Published:** 2022-06-02

**Authors:** Yinzhong Shen, Jingwen Ai, Na Lin, Haocheng Zhang, Yang Li, Hongyu Wang, Sen Wang, Zhen Wang, Tao Li, Feng Sun, Zhenyu Fan, Liqun Li, Yunfei Lu, Xianmin Meng, Hong Xiao, Huiliang Hu, Yun Ling, Feng Li, Hongdi Li, Chunmei Xi, Liping Gu, Wenhong Zhang, Xiaohong Fan

**Affiliations:** a Department of Infection and Immunity, Shanghai Public Health Clinical Centre, Fudan University, Shanghai, People’s Republic of China; b Department of Infectious Diseases, National Medical Centre for Infectious Diseases, National Clinical Research Centre for Aging and Medicine, Shanghai Key Laboratory of Infectious Diseases and Biosafety Emergency Response, Huashan Hospital, Fudan University, Shanghai, People’s Republic of China; c Department of COVID-19, Shanghai Public Health Clinical Centre, Fudan University, Shanghai, People’s Republic of China; d Lingang Laboratory, Shanghai, People’s Republic of China; e Department of Tuberculosis, Shanghai Public Health Clinical Centre, Fudan University, Shanghai, People’s Republic of China; f Department of Infectious Diseases, Shanghai Public Health Clinical Centre, Fudan University, Shanghai, People’s Republic of China; g Department of Respiratory Diseases, Shanghai Public Health Clinical Centre, Fudan University, Shanghai, People’s Republic of China; h National Clinical Research Centre for Aging and Medicine, Huashan Hospital, Fudan University, Shanghai, People’s Republic of China; i Key Laboratory of Medical Molecular Virology (MOE/MOH), Shanghai Medical College, Fudan University, Shanghai, People’s Republic of China

**Keywords:** Omicron variant, COVID-19, VV116, cohort study, viral load

## Abstract

Omicron variant of SARS-CoV-2 has become the predominant variant worldwide. VV116 is an oral drug with robust anti-SARS-CoV-2 efficacy in preclinical studies. We conducted an open, prospective cohort study to evaluate its safety and effectiveness in Chinese participants infected with the omicron variant from March 8th, 2022 to March 24th, 2022. 136 hospitalized nonsevere patients confirmed with COVID-19 were enrolled including 60 patients who received VV116 (300 mg, BID×5 days) in the treatment group and 76 patients who didn’t receive VV116 in the control group besides standard treatment. Viral load shedding time and adverse events were collected during the follow-up. There was no significant difference in baseline characteristics between the VV116 group and the control group, except for a higher symptom prevalence in the control group (*P* = 0.021). The median time from the first positive test to the first VV116 administration was 5 (range: 2-10) days. Participants who received VV116 within 5 days since the first positive test had a shorter viral shedding time than the control group (8.56 vs 11.13 days), and cox regression analysis showed adjusted HR of 2.37 [95%CI 1.50-3.75], *P *< 0.001. In symptomatic subgroup, VV116 group had a shorter viral shedding time than the control group (*P *= 0.016). A total of 9 adverse events with no serious adverse events were reported in the VV116 group, all of them were resolved without intervention. VV116 is a safe, effective oral antiviral drug, which shows a better performance within the early onset of omicron infection.

## Introduction

The coronavirus disease 2019 (COVID-19) pandemic is one of the greatest threats to human health in the twenty-first century and causes a significant impact on human life and human society. Currently, the omicron variant of SARS-CoV-2 has become the predominant variant circulating in most countries and leads to increased vaccine breakthrough rates and widespread escape from existing neutralizing antibodies due to high mutations in the spike protein, the target of most COVID-19 vaccines and therapeutic antibodies [1,2]. Small molecule SARS-CoV-2 antiviral drugs generally targeting the conserved viral RNA-dependent RNA polymerase (RdRp) or the conserved viral main protease rather than the spike protein are unlikely to lose activity against variants. Two oral antiviral drugs, nirmatrelvir/ritonavir (Paxlovid) and molnupiravir have received the FDA’s emergency use authorization (EUA) for the treatment of mild to moderate COVID-19 outpatients who are at risk for progression. Both nirmatrelvir and molnupiravir retain their activity against all current variants of concern including Omicron [3,4]. However, the low clinical efficacy and possible mutagenic effects of molnupiravir and the potential for significant drug–drug interactions of Paxlovid limited their widespread clinical use. With increasing numbers of cases and deaths worldwide and the very limited treatment options, there is still an immediate unmet medical need for new effective oral antiviral drugs in the control of COVID-19.

VV116 is a new effective oral nucleoside drug candidate against SARS-CoV-2, which could inhibit SARS-CoV-2 replication, lower viral RNA levels and infectious virus titres *in vivo* and *in vitro*, along with satisfying tolerability safety and pharmacokinetic profile in 3 phase 1 studies [5,6]. VV116 targets the conserved viral RdRp, and demonstrated potent antiviral activities against the omicron variant with an EC_90_ of 0.30 μM, comparable to that of the original strain (EC_90_: 0.83 μM). VV116 has been approved for the treatment of COVID-19 in Uzbekistan and is being investigated in several Phase 3 clinical trials in patients with COVID-19 (ClinicalTrials.gov Identifier: NCT05242042, NCT05279235, NCT05341609). In this article, we report the open, prospective cohort study of VV116 in Chinese participants infected with SARS-CoV-2 omicron variant.

## Methods

### Objectives, patients, and oversight

This is an open, prospective cohort study to evaluate the safety and viral clearance (real-time PCR Ct value>35 for both ORF1ab and N gene) during clinical administration of VV116. Eligible patients were required to be at least 18 years old with confirmed SARS-CoV-2 infection by real-time PCR from March 8th to March 24th, 2022. Exclusion criteria included: (1) Patients who were diagnosed as severe or critical COVID-19 before intervention; (2) SpO_2_<93%, or PaO_2_/FiO_2_≤300 mmHg, or respiratory rate≥30 breaths/min, or heart rate≥125 beats/min. (3) Patients who needed mechanical ventilation; (4) patients who had been confirmed with active bacterial, fungal or viral infection besides COVID-19; (5) patients with eye disease including inflammation, vascular malformation, retinal haemorrhage, etc. (6) HIV infection; (7) Alanine transaminase or aspartate transaminase > 1.5 upper limit of normal; (8) allergic to any ingredients of VV116; (9) patients who had received SARS-CoV-2 monoclonal antibody, or antiviral treatment, or convalescent plasma; (10) patients who or whose partner were pregnant, lactating, or refused to comply with the contraception requirements; (11) patients who had been engaged into other interventional clinical trial.

This study has been approved by the local ethic committee with an IRB of 2022-E018-01. Informed consent was obtained from all participants before enrolment and drug administration.

### Procedures

From March 8th to March 24th, after receiving consents from the participants, demographic information, disease history, vital signs, disease condition, and Ct values were collected for each participant at the baseline. After a detailed introduction to the trial and VV116, patients who agreed to receive VV116 for treatment would be enrolled into the VV116 group and patients who rejected VV116 but agreed to participate would be enrolled into the control group. Informed consents were collected from all the participants. In the VV116 group, participants would receive 300mg of VV116 orally every 12 h for 5 days (10 doses total). Vital signs and disease conditions were monitored by clinicians every day and the treatment courses were retrospectively summarized at the end of follow-up. All participants received standard treatment according to the guideline.

### Viral clearance

The primary endpoint was time to viral clearance. SARS-CoV-2 viral load was detected and quantified by RT–PCR using nasopharyngeal swabs on alternate days since hospital admission. If the nucleic acid test was still positive for COVID-19 for a participant, SARS-CoV-2 viral load was further quantified. Ct value>35 for both ORF1ab and N gene was considered as negativity. The nucleic acid test negative conversion was defined as two consecutive negative tests (Ct value>35 for the ORF1ab and N gene). The viral shedding time was defined as first positive nucleic acid test to the date of the first negative test (in two consecutive).

### Safety

The safety endpoint was to evaluate the adverse events during and post drug administration, including overall and each incidence of adverse events, serious adverse events, as well as discontinuation of drug administration due to adverse events. Safety data were collected only after participants received the first dose of VV116 in the VV116 group.

### Statistical analysis

Continuous variables were expressed as median (Range) or mean (Standard Deviation, SD) and compared with the non-parametric test. Categorical variables were expressed as number (%) and compared by the χ² test or Fisher’s exact tests.

The primary analysis and the subgroup analysis compared the viral shedding time between the two groups. Hazard ratio (HR) and 95% confidence interval were calculated by Cox regression. A two-sided *P *< 0·05 was considered statistically significant.

## Result

Between March 8th and March 24th, 2022, 136 hospitalized patients with confirmed COVID-19 were enrolled in this study, including 60 in the VV116 group and 76 in the control group. Participant characteristics were generally similar between the two groups (Table 1) except for the symptom prevalence. In all participants enrolled, the mean age was 33.9 years; 92 patients (67.6%) were male. A higher proportion of participants reported symptoms in the control group than in the VV116 group (51.3% vs. 31.7%, *p* = 0.021). The most frequently reported symptoms were cough (46 participants, [33.8%]) and sputum production (36 participants, [33.8%]) in all participants enrolled. Comorbidities were not common in both groups. Vaccination status between the two groups was comparable, and among all participants, 11.0% were unvaccinated, 0.7% were partially vaccinated with one dose, 45.6% were fully vaccinated by two doses, and 42.6% received a third booster dose.

**Table 1.** Baseline characteristics.

	VV116 (*N* = 60)	Control (*N* = 76)	*P* value
Age, Mean ± SD, years	34.4 ± 21.0	33.5 ± 17.2	0.574
Male, *n* (%)	45 (75.0)	47 (61.8)	0.103
Hypertension, *n* (%)	1 (1.7)	5 (6.6)	0.228
Diabetes, *n* (%)	2 (3.3)	0 (0)	0.193
Symptoms, *n* (%)			0.021
Prevalence of symptoms	19 (31.7)	39 (51.3)	
Fever	8 (13.3)	9 (11.8)	
Cough	15 (25.0)	31 (40.8)	
Sputum production	13 (21.7)	23 (30.3)	
Sore throat	3 (5.0)	5 (6.6)	
Muscle pains	6 (10.0)	6 (7.9)	
Fatigue	5 (8.3)	5 (6.6)	
Vaccination, *n* (%)			0.090
Unvaccinated	3 (5.0)	12 (15.8)	
Partially vaccinated	0 (0.0)	1 (1.3)	
Full vaccination	26 (43.3)	36 (47.4)	
Booster	31 (51.7)	27 (35.5)	
Initial SARS-CoV-2 RT–PCR tests			
ORF, Mean ± SD	25.2 ± 6.62	26.9 ± 6.02	0.262
N, Mean ± SD	26.6 ± 6.50	23.7 ± 6.79	0.063
Administration time since the first positive test			
≤5 days, n (%)	32 (53.3)	/	
>5 days, n (%)	28 (46.7)	/	
Median (range), days	5 (2-10)	/	
Mean ± SD, days	5.8 ± 1.8	/	

Abbreviation: SD, standard deviation.

Continuous variables were compared with the non-parametric test. Categorical variables were compared by the χ² test or Fisher’s exact tests.

Most patients were mild cases, and the percentage of moderate cases was 5.0% (3/60) and 5.2% (4/76) in the VV116 group and the control group, respectively. None were severe cases in this study. All participants received symptomatic treatment according to clinical manifestations and laboratory examinations, including non-steroidal anti-inflammatory drugs and cough mixtures. None of participants received other antiviral drugs, corticosteroids, or monoclonal antibody. In the VV116 group, 5 (8.3%) participants were treated within 3 days after the first positive test for SARS-CoV-2, with a median duration of 5 days.

A total of 9 (15.0%) adverse events with no serious adverse events were reported among the VV116 group, 7 (11.6%) of which were mild liver function abnormalities (3 elevated alanine aminotransferase, 3 elevated indirect bilirubin, and 1 elevated bile acid), and all of them were resolved without further intervention. The less frequently reported adverse events were elevated blood urea (1/60, 1.7%) and elevated white blood cell count (1/60, 1.7%).

**Figure 1.**
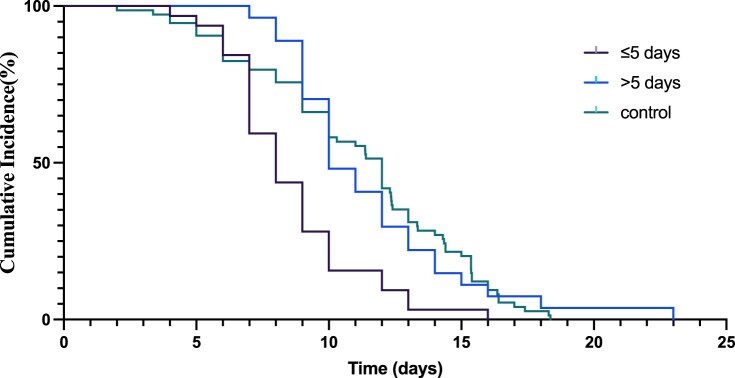
The Kaplan–Meier estimate of the time from the first positive testing to the first day of nucleic acid Ct value >35 for both ORF1ab and N gene.

In all participants enrolled in the study, the SARS-CoV-2 nucleic acid shedding time was 9.92 (95%CI: 9.06–10.77) days and 11.13 (95%CI: 10.22–12.04) days in the VV116 group and the control group, respectively. Time from the first day of VV116 treatment to the first day for the negative nucleic acid testing is 3.52 (95% CI 2.83–4.23) days. We then evaluated the shedding time based on drug administration within or more than 5 days since the first positive test (≤5 days group and >5 days group) in the VV116 group. The SARS-CoV-2 nucleic acid shedding time was 8.56 (95%CI: 7.64–9.48) days in the ≤5 days group and 11.46 (95%CI: 10.13–12.80) days in the >5 days group, respectively. The shedding time was shorter in the ≤5 days group than in the control group (adjusted Hazard Ratio, 2.37, 95% confidence interval [CI], 1.50–3.75; *P* < 0.001) (Figure 1, Table 2, Table 3). However, no significant difference was found between the >5 days group and the control group.

**Table 2.** The hazard ratios, two-sided 95% confidence intervals, and *P* value were estimated with the use of Cox regression with the baseline stratification factors as covariates.

	Adjusted hazard ratio	*P* value
Administration time since the first positive test		0.000
Control	Ref	
≤5 days	2.37(1.50, 3.75)	0.000
>5 days	0.77(0.48, 1.25)	0.286
Age	0.99(0.98, 1.00)	0.098
Gender		0.889
Male	Ref	
Female	1.03(0.70, 1.51)	
Symptoms		0.014
symptomatic	Ref	
asymptomatic	1.59(1.10, 2.31)	
Vaccination		0.966
Unvaccinated	Ref	
Partially vaccinated	0.85(0.11, 6.67)	0.877
Full vaccination	1.12(61, 2.05)	0.716
Booster	1.15(0.62, 2.11)	0.659

**Table 3.** Subgroup analysis of the differences in SARS-CoV-2 nucleic acid shedding time using the non-parametric test.

Subgroups (95% CI)	VV116 (*N* = 60)	Control (*N* = 76)	*P* value
Administration time since the first positive test			
≤5 days	8.56 (7.64,9.48) (*N* = 32)	11.13 (10.22, 12.04) (*N* = 76)	0.001
>5 days	11.46 (10.13,12.80) (*N* = 28)	11.13 (10.22, 12.04) (*N* = 76)	0.846
Gender			
Female	9.93 (7.79,11.88) (*N* = 15)	11.15 (9.66,12.64) (*N* = 29)	0.339
Male	9.91 (8.92,10.90) (*N* = 45)	11.12 (9.93,12,31) (*N* = 47)	0.056
Symptoms			
asymptomatic	9.88 (8.75,11.00) (*N* = 41)	9.95 (8.69,11.20) (*N* = 37)	0.669
symptomatic	10.00 (8.64,11.35) (*N* = 19)	12.25 (10.99,13.51) (*N* = 39)	0.016
Vaccination			
Unvaccinated	9.33 (−0.71,19.37) (*N* = 3)	13.05 (11.34,14.76) (*N* = 12)	0.136
Partially vaccinated	/ (*N* = 0)	12.00 (*N* = 1)	/
Full vaccination	10.42 (8.99,11.86) (*N* = 26)	10.46 (9.07,11.85) (*N* = 36)	0.758
Booster	9.55 (8.41,10.68) (*N* = 31)	11.13 (9.48,12.79) (*N* = 27)	0.080

^a^ Time since the first positive testing to the first day of nucleic acid Ct value >35 for both ORF1ab and N gene.

Continuous variables were compared with the non-parametric test.

In the subgroup analyses, the shedding time of the symptomatic groups was shorter in the VV116 group (10.00 [95%CI: 8.64–11.35] days) than in the control group (12.25 [95%CI: 10.99–13.51] days) with a statistically significant difference (*P *= 0.016). (Table 3).

## Discussion

This real-world study evaluated the viral load shedding time and adverse events of SARS-CoV-2 omicron variant-infected participants treated with VV116, a new oral antiviral drug. The overall viral shedding time was 9.92 and 11.13 days in the VV116 group and the control group, respectively (*P* > 0.05). Participants who received VV116 within 5 days since the first positive test had a shorter viral shedding time than the control group, while there was no significant difference between the >5 days group and the control group. Symptomatic patients had a shorter viral shedding time in the VV116 group compared with the control group. No serious adverse events were reported in both groups.

Between 26 February and 23 April 2022, Shanghai had reported 42,379 confirmed cases and 370,320 asymptomatic carriers, among whom 175 (0.4%) were severe or critical cases and 55 (0.1%) people died [7]. Compared to 50,354 confirmed cases with a 63% severity rate and 7.7% death rate as reported in Wuhan in 2020 [8], the total number in this period in Shanghai was much higher while the ratio of severity and mortality was much lower. A similar phenomenon has also been observed in other countries. Between November 11 2021 and January 5 2022 in Huston, Texas, where omicron (B.1.1.529) has been proved to cause 98% of new cases, 56.4% of cases required at least low-flow oxygen, which was significantly lower than cases caused by alpha or delta variant [1]. Previous reports and all data above suggested that omicron caused more infections but less severe cases or death, while continuous outbreaks and the huge population base still made the medical system overloaded and exhausted. Shortening the disease duration and preventing severe illness with VV116 could be another option for us to fight against this disease.

Indications and intervention time are two of the most crucial parameters for oral antivirals usage. Although Remdesivir, GS-441524, molnupiravir, and nirmatrelvir have shown similar inhibition effects on viral replication *in vitro* experiment [3], the clinical trials presented to be significantly different. Remdesivir was authorized by FDA in 2020 for cases who required hospitalization, while drug studies did not report statistically significant clinical benefits in these relatively severe cases [9–11]. In the phase 2 study of molnupiravir, clinical benefit and reduction of virus load were observed only in participants treated ≤5 days from symptom onset. Molnupiravir and Paxlovid were indicated for un-hospitalized cases with high risks for severe illness within 5 days after symptom onset and proved to be effective [12,13]. VV116 is an oral drug with robust anti-SARS-CoV-2 efficacy in preclinical studies and satisfactory safety, tolerability, and pharmacokinetic properties in healthy Chinese subjects [5,6]. In this study, VV116 significantly accelerated the viral shedding by 2–3 days when applied at the early stage of disease. Our study implied that VV116 might benefit patients treated within 5 days since the first positive nucleic acid results, and therefore VV116 should be administered earlier. In addition, subgroup analysis revealed that in symptomatic participants VV116 shortened the time to viral clearance regardless of the first time of drug administration. The administration time since the first positive test was within 2–10 days with a median of 5 days. Therefore, for symptomatic cases, administration of VV116 may be helpful within 10 days since the first positive test. Furthermore, our study enrolled patients who received full-course vaccination or booster vaccination in both the VV116 and control groups, which implied that VV116 might still possibly benefit patients who have been previously vaccinated.

Variants of omicron in receptor binding domain (RBD) region led to tighter RBD/ACE2 interaction and enhanced ability to escape from immune responses [2]. Thus, both convalescent serum and most monoclonal antibodies were reduced or even failed to neutralize omicron variants *in vitro* experiments [2,4,14], while small molecule antivirals had been proved to be still effective against these variants of concern [3]. Omicron variant carried only one mutation in nsp12, and the changed amino acid was distant from the active site of RdRp, which may explain why VV116 maintained activity against omicron variant. Paxlovid (nirmatrelvir/ritonavir) remained to be effective against Omicron in VeroE6-GFP cells and has been proved to be 89% effective in patients at risk of serious illness [3,12], which lower the hospitalization rate by 5.81% and accelerate the clearance of viral load in a phase 2–3 double-blind, randomized, controlled trial [15].

Previously published studies showed an overall satisfactory safety profile among patients who received nirmatrelvir plus ritonavir, the most common adverse events were dysgeusia (5.6%) and diarrhoea (3.1%), other adverse events included fibrin D-dimer increase, liver enzyme increase, headache, and nausea. Most of these adverse events were mild grade 1 or 2 and could resolve by themselves quickly, and few participants reported serious adverse events such as decreased renal creatinine or COVID-19 pneumonia. No serious adverse events were found in all enrolled participants in this study. The frequency of liver injury was comparable with other studies [16,17]. Overall, VV116 showed satisfactory safety results.

This study had several limitations. First, administration of VV116 or not was based on clinical practice rather than randomization. Second, we adopted a viral shedding time from first positive nucleic acid test to fit both the VV116 group and the control group, thus not all the patients were diagnosed on the first day of their viral shedding. Another limitation was the sample size. What’s more, the baseline of the control group and VV116 group has no significant difference except that the rate of the symptomatic patients was higher in the control group, thus in the study we have used the cox-hazard model to adjust the potential bias of the baseline. However, this limitation’s potential influence should be acknowledged and further phase 2/3 randomized clinical trials are required in the future.
